# Do GPs know their patients with cancer? Assessing the quality of cancer registration in Dutch primary care: a cross-sectional validation study

**DOI:** 10.1136/bmjopen-2016-012669

**Published:** 2016-09-15

**Authors:** Annet Sollie, Jessika Roskam, Rolf H Sijmons, Mattijs E Numans, Charles W Helsper

**Affiliations:** 1 Department of General Practice & Elderly Care Medicine, VU University Medical Centre, Amsterdam, The Netherlands; 2 Julius Centre for Health Sciences and Primary Care, University Medical Centre Utrecht, Utrecht, The Netherlands; 3 Department of Genetics, University of Groningen, University Medical Centre Groningen, Groningen, The Netherlands; 4 Department of Public Health and Primary Care, Leiden University Medical Centre, Leiden, The Netherlands

**Keywords:** PRIMARY CARE

## Abstract

**Objectives:**

To assess the quality of cancer registry in primary care.

**Design and setting:**

A cross-sectional validation study using linked data from primary care electronic health records (EHRs) and the Netherlands Cancer Registry (NCR).

**Population:**

290 000 patients, registered with 120 general practitioners (GPs), from 50 practice centres in the Utrecht area, the Netherlands, in January 2013.

**Intervention:**

Linking the EHRs of all patients in the Julius General Practitioners’ Network database at an individual patient level to the full NCR (∼1.7 million tumours between 1989 and 2011), to determine the proportion of matching cancer diagnoses. Full-text EHR extraction and manual analysis for non-matching diagnoses.

**Main outcome measures:**

Proportions of matching and non-matching breast, lung, colorectal and prostate cancer diagnoses between 2007 and 2011, stratified by age category, cancer type and EHR system. Differences in year of diagnosis between the EHR and the NCR. Reasons for non-matching diagnoses.

**Results:**

In the Primary Care EHR, 60.6% of cancer cases were registered and coded in accordance with the NCR. Of the EHR diagnoses, 48.9% were potentially false positive (not registered in the NCR). Results differed between EHR systems but not between age categories or cancer types. The year of diagnosis corresponded in 80.6% of matching coded diagnoses. Adding full-text EHR analysis improved results substantially. A national disease registry (the NCR) proved incomplete.

**Conclusions:**

Even though GPs do know their patients with cancer, only 60.6% are coded in concordance with the NCR. Reusers of coded EHR data should be aware that 40% of cases can be missed, and almost half can be false positive. The type of EHR system influences registration quality. If full-text manual EHR analysis is used, only 10% of cases will be missed and 20% of cases found will be wrong. EHR data should only be reused with care.

**Strengths and limitations of this study**

- Use of record linkage between patient electronic health records (EHRs) and Netherlands Cancer Registry to assess the quality of cancer registry in primary care.
- Size of cohort (290 000 patients) and availability of extensive, real routine-care EHR data from general practices.
- Includes study for causes of inadequate registry and resulting opportunities for improvement.
- Since the study was performed in the Netherlands, results are indicative for countries with a similar primary care setting.
- Only a sample of false-positive and false-negative cases could be analysed to find reasons for inadequate registry of cancer diagnosis.

## Introduction

Ask general practitioners (GPs) if they know their patients with cancer and they will most likely answer with an outspoken ‘yes’! Ask them if these patients are registered with this cancer diagnosis in their electronic health record (EHR) system and the answer will be ‘yes, probably’. Most GPs will acknowledge the importance of adequate disease registry in EHRs, certainly for a serious disease such as cancer, since these records are used for information exchange between care providers.

Since reuse of EHRs for other purposes such as chronic disease management,^1^ research^2–4^ and quality assessment^5^ ^6^ is becoming commonplace, not only in hospitals^7^ ^8^ but also in primary care,^2–4^ correct and complete registry of diagnoses using coding systems is pivotal.^9–11^ Disease registry using coding systems has been common practice in primary care EHRs for almost two decades in Western countries. In several countries including the Netherlands, guidelines have been developed for correct registration in the EHR that do address adequate disease coding.^12^

Despite these developments, there are indications that (coded) disease registry in primary care is still suboptimal,^13^ ^14^ even for important diagnoses such as cancer.^15^ The literature describing the quality of data in primary care however is limited. To assess quality and subsequent (re)usability of EHR data, it is important to quantify this quality and to determine which variables influence the quality of disease registry. We will assess various aspects of the quality of disease registry in primary care for reuse purposes. We focus on cancer since supposedly reliable and elaborate information concerning cancer diagnoses is available from the Netherlands Cancer Registry (NCR), thereby providing a potential reference standard required for our study.

We aim to answer the following questions:

1. What are the proportions of matching, missing (potentially false-negative) and wrong (potentially false-positive) cancer diagnoses in the primary care EHR using the NCR as a reference standard?
2. How accurate is the year of diagnosis registered in the EHR for the matching cancer cases, when compared to the NCR as a reference standard?
3. Do age of the patient, cancer type and EHR system influence the quality of cancer diagnosis registry?
4. What are the causes of suboptimal cancer diagnosis registry in the EHR and subsequent opportunities for improvement?

## Methods

### Design

Using a cross-sectional validation study, we assessed the proportion of matching, missing (potentially false-negative) and wrong (potentially false-positive) breast, lung, colorectal and prostate cancer diagnoses in primary care EHR data between 2007 and 2011, using the NCR as a reference standard. We linked the EHR to the NCR at an individual patient level using a trusted third party (TTP), to obtain an anonymous data set containing the EHR and the NCR data. We defined coded diagnoses representing the same cancer type in both databases as matching cases. We defined missing (potentially false-negative) diagnoses as occurring with one or more of the four cancers under study in the NCR, but not in the EHR in one of the years 2007–2011. We defined wrong (potentially false-positive) diagnoses as registered with one of the four cancer types under study in the EHR, but not registered with the same diagnosis in the NCR, in one of the years 2007–2011.

### Data

We used the routine EHR data extracted from practice centres in the Utrecht area, the Netherlands, that are a member of the Julius General Practitioners' Network (JGPN; 120 GPs, 50 practice centres, 290 000 patients). Coded and free-text primary care data from individual patients enlisted with these centres are periodically extracted to the central anonymised EHR JGPN database. Data were included if GPs used one of the three most frequently used EHR systems in the study region: Promedico, Medicom and MicroHis. These systems cover 85% of the population registered with participating GPs. The systems vary in design and user interface but are all based on the reference model provided by the Dutch College of General Practitioners. The JGPN population is considered representative of the Dutch population,^16^ and its GPs and GP centres represent the average Dutch GPs and GP centres. GPs were not aware of this study at the time of registry; neither did they receive specific training on coding. Hence, the data in the JGPN can be regarded as true ‘routine care data’.

In the Netherlands, GP medical encounters are registered according to the ‘SOAP-system’.^17^ A SOAP-journal consists of four data fields. The first is ‘subjective’ (S) and is used to register in plain text what the patient describes, such as symptoms and the reason for the encounter. The second data field is called ‘objective’ (O) and includes the GP's findings and results from clinical examination and measurements in plain text. The third field is ‘analysis’ (A), which is used to register the (working) diagnosis, most important symptom or hypothesis as plain text and is coded using the International Classification of Primary Care V.1 (ICPC-1) coding system.^18^ The final field is ‘plan’ (P), comprising the GPs medication prescriptions, diagnostic tests, referrals to medical specialists and follow-up appointments as plain text. The list ‘episodes’, also coded using ICPC-1, clusters consultations concerning the same diagnosis for an individual patient with corresponding start and end dates. According to the Dutch College of General Practitioners' guideline^12^ for correct registration, every cancer diagnosis should be registered as an episode in the EHR and consultations concerning relevant symptoms or treatments should be added to this episode. The guideline also states that it is mandatory for GPs to update the EHR episode with the final diagnosis.

ICPC diagnosis codes are available for the more common types of cancer, including cancers under study. There are no separate codes available for the recurrence of cancer, for suspected cancer or for treatments of cancer. The GP manually enters the ICPC code for a ‘cancer’ diagnosis during consultation or after receiving secondary care correspondence. A diagnosis code should only be used in the EHR after confirmation of the diagnosis and not if a diagnosis is suspected. The GP decides if and when a new episode is created for the cancer diagnosis and which consultations are added to this episode.

### Reference standard

Elaborate information on cancer diagnoses and treatment is available in the NCR.^19^ Specially trained staff members enter relevant data about all Dutch cancer diagnoses in the NCR database, triggered by hospital pathology reports of newly found cancers. In addition, cancer diagnoses reported in hospital patient discharge files, for which no pathological investigation is being performed, are also included in the NCR as clinical diagnoses for most hospitals. The NCR claims to be almost complete (>95% of all cancers) for the population of the Netherlands and without false-positive records since 1989.

There is a registration delay reported at the NCR of 3–9 months after the pathologist confirmed cancer, and the delay is claimed to be decreasing. There is some evidence that the quality of the NCR data is complete and accurate.^20^ ^21^ Theoretically, patients with cancer missing in the NCR could include those who are diagnosed with cancer in primary care based on clinical signs and symptoms, but are unable or refuse to go to a hospital. Therefore, for these patients, no pathology report or hospital admission is registered, which would be needed to enter the NCR.

### Data collection and analysis

#### Step 1: identification of cancer cases in primary care EHR

We identified all breast, lung, colorectal and prostate cancer cases diagnosed at ages 20–90 between 2007 and 2011 in the EHR, using a three-step search strategy. First, we searched for patients with one or more cancer episodes in the database with ICPC codes X76 (breast cancer), R84 (lung cancer), D75 (colorectal cancer) and/or Y77 (prostate cancer). Next, we selected all cases without a coded cancer-related episode but with one or more encounters (home visits, correspondence, consultation) coded with X76, R84, D75 and/or Y77. Finally, we selected patients without an episode or medical encounter coded for the types of cancer under study, but with whom any prescription for cancer-specific medication was registered during the observation time. After identifying these patients, a subset of data including all the required information was extracted from the EHR: ICPC code, year of diagnosis, age at diagnosis, year of birth, sex and type of EHR system used.

#### Step 2: linkage of data

Parallel to step 1, the TTP linked the entire JGPN database with the entire NCR database. The linking was performed after encryption of the data using a mixed algorithm with deterministic and probabilistic parts based on the following variables: date of birth, gender, zip code, last name, initials and first name. If the date of birth and gender matched (deterministic part), the probabilistic part of the algorithm started based on the Fellegi-Sunter^22^ model. This means the other variables were compared, yielding scores, which were totalled and evaluated using weights.

The TTP provided a list with pseudonymised patient numbers of all patients that were successfully linked and added to the NCR data. JGPN data management added EHR data to every patient number on the list.

#### Step 3: matching diagnoses

Dutch GPs use the ICPC-1 coding system. The NCR uses the International Classification of Diseases for Oncology (ICD-O). Diagnoses were counted as matching when their ICPC-1 code and ICD-O code represented the same cancer (table 1).

**Table 1:** Cross-linking of ICPC-1 with ICD-O codes used to define matching cancers in the electronic health records and the Netherlands Cancer Registry

Cancer type	ICPC-1 codes used in electronic health record	ICD-9/10-O codes used in Netherlands cancer registry
Colorectal cancer	D75 incl. subtypes:	C18*
	D75.01	C19*
	D75.02	C20*
	D75.03	153†
		154†
Lung cancer	R84	C34
		162†
		163†
Breast cancer	X76 incl. subtypes:	C50*
	X76.1	174†
	X76.01	
	X76.02	
	X76.03	
Prostate cancer	Y77	C61*
		158†

*Codes from ICD-10 (since 1990).

†Codes from ICD-9 (since 1978).

ICD-O, International Classification of Diseases for Oncology (ICD-O); ICPC-1, International Classification of Primary Care V.1.

Diagnoses were stratified based on cancer type, age category (<50, 50–75 and >75) and EHR system. Differences in the year of diagnosis between EHR and NCR were determined by subtracting the year of diagnosis at the GP from the year of diagnosis as registered in the NCR.

#### Step 4: non-matching (missing and wrong) diagnoses

Non-matching diagnoses were assessed using the NCR as a reference. We determined the proportion of missing (potentially false-negative) diagnoses in the EHR and stratified these per cancer type and per age category. We also determined the proportion of wrong (potentially false-positive) diagnoses stratified by cancer type, age category and EHR systems used. In case of repeated entries of a new diagnosis for the same person in the EHR, we counted one as being correct and the other(s) as false positive.

We extracted and studied the full EHR of a random sample of 120 of the 1644 (7.3%) potentially false-positive EHR diagnoses and 120 of the 1720 (7.0%) potentially false-negative EHR diagnoses to determine reasons for inaccurate and incomplete GP disease registry. To ensure representativeness of our sample, we used the following sampling method: the sample of false-positive cases consisted of 4×30 cases per cancer type equally distributed over the years of interest and the EHR system used. The sample of false-negative cases consisted of five cases per cancer type per year. The EHR system could not be taken into account in the selection of false-negative cases since this could only be determined after extracting the full EHR of the sample.

All data analysis and calculations were carried out using SPSS Statistics V.21.

### Patient involvement

Patients were not involved in the design, development of outcome measures or conduct of this study. Since this study uses anonymised EHR data from an existing network database only, no patients were recruited and thanking patients or disseminating results directly is not applicable.

## Results

The linkage by the TTP of the full JGPN database to the full NCR database yielded 12 930 JGPN participants with a registered cancer at the NCR (data from January 2013), of whom 12 526 could be included in our analysis. The remaining 404 (3.1%) records belonged to 202 patients who were matched twice. These records were considered incorrectly identified. We had to remove another 14 (0.1%) records for suspected wrong linkage before starting our analyses.

The extraction of breast, lung, colorectal and prostate cancer diagnoses yielded 3364 cases from the EHR data and 2839 from the NCR (table 2).

**Table 2:** Results of record linking between the electronic health records and the Netherlands Cancer Registry

	Total cancers JGPN	Total cancers NCR	Number matching	Proportion matching		Number false positives	Proportion false positives		Number false negatives	Proportion false negatives	
	n	m	N	N/m (%)	95% CI	M	M/n (%)	95% CI	K	K/m (%)	95% CI
Four types combined	3364	2839	1720	60.6	(58.8 to 62.4)	1644	48.9	(47.2 to 50.6)	1119	39.4	(37.6 to 41.2)
Age <50	451	412	246	59.7	(55.0 to 64.4)	205	45.5	(40.9 to 50.1)	166	40.3	(35.5 to 45.0)
Age 50–75	2109	1824	1139	62.4	(60.2 to 64.7)	970	46.0	(43.9 to 48.1)	685	37.6	(35.3 to 39.8)
Age >75	804	603	335	55.6	(51.6 to 59.5)	469	58.3	(54.9 to 61.7)	268	44.4	(40.5 to 48.4)
EHR system											
Promedico	2068	x	925	44.7		1143	55.3	(53.1 to 57.4)	x		
Medicom	468	x	261	55.8		207	44.2	(39.7 to 48.7)	x		
MicroHis	828	x	534	64.5		294	35.5	(32.2 to 38.8)	x		
Cancer type											
Breast cancer	1144	1008	622	61.7	(58.7 to 64.7)	522	45.6	(42.7 to 48.5)	386	38.3	(35.3 to 41.3)
Age <50	290	267	156	58.4	(52.5 to 64.3)	134	46.2	(40.5 to 51.9)	111	41.6	(35.7 to 47.5)
Age 50–75	681	598	381	63.7	(59.7 to 67.6)	300	44.1	(40.3 to 47.8)	217	36.3	(32.4 to 40.1)
Age >75	173	143	85	59.4	(51.4 to 67.5)	88	50.9	(43.4 to 58.3)	58	40.6	(32.5 to 48.6)
Prostate cancer	662	547	336	61.4	(57.3 to 65.5)	326	49.2	(45.4 to 53.1)	211	38.6	(34.5 to 42.7)
Age <50	6	5	4	80.0	(44.9 to 115.1)	2	33.3	(−4.4 to 71.1)	1	20.0	(−15.1 to 55.1)
Age 50–75	469	425	270	63.5	(59.0 to 68.1)	199	42.4	(38.0 to 46.9)	155	36.5	(31.9 to 41.0)
Age >75	187	117	62	53.0	(43.9 to 62.0)	125	66.8	(60.1 to 73.6)	55	47.0	(38.0 to 56.1)
Lung cancer	731	600	331	55.2	(51.2 to 59.1)	400	54.7	(51.1 to 58.3)	269	44.8	(40.9 to 48.8)
Age <50	46	36	18	50.0	(33.7 to 66.3)	28	60.9	(46.8 to 75.0)	18	50.0	(33.7 to 66.3)
Age 50–75	492	438	248	56.6	(52.0 to 61.3)	244	49.6	(45.2 to 54.0)	190	43.4	(38.7 to 48.0)
Age >75	193	126	65	51.6	(42.9 to 60.3)	128	66.3	(59.7 to 73.0)	61	48.4	(39.7 to 57.1)
Colon cancer	827	684	431	63.0	(59.4 to 66.6)	396	47.9	(44.5 to 51.3)	253	37.0	(33.4 to 40.6)
Age <50	74	58	43	74.1	(62.9 to 85.4)	31	41.9	(30.7 to 53.1)	15	25.9	(14.6 to 37.1)
Age 50–70	502	409	265	64.8	(60.2 to 69.4)	237	47.2	(42.8 to 51.6)	144	35.2	(30.6 to 39.8)
Age >75	251	217	123	56.7	(50.1 to 63.3)	128	51.0	(44.8 to 57.2)	94	43.3	(36.7 to 49.9)

JGPN, Julius General Practitioners’ Network; NCR, Netherlands Cancer Registry.

Overall, 60.6% (1720 of 2839) of cases matched (‘sensitivity’ of the EHR), which means that 39.4% (1644 of 2839) of cancer cases seem to be missing in the EHR (potentially false negative). Furthermore, 1644 (48.9% of 3364) of EHR cases were not found in the NCR, thus should be qualified as potentially false positive. Consequently, the ‘positive predictive value’ of a cancer diagnosis in the EHR is 51.1%. The two by two table illustrates these findings, including a negative predictive value of 99.5% and a specificity of 99.3% (table 3).

**Table 3:** Two by two table for matching and non-matching cancer cases registered from 2007 to 2011 in the JGPN and NCR

	Cancer status according to NCR		
Cancer status according to JGPN	Cancer present	Cancer absent	Total
Cancer present	1720	1644	3364
Cancer absent	1119	237 906	239 025
Total	2839	239 550	242 389*

*Population of the JGPN registered in the included EHR systems.

EHR, electronic health record; JGPN, Julius General Practitioners’ Network; NCR, Netherlands Cancer Registry.

We found no substantial differences in proportion of matching, potentially false-negative and false-positive cases between cancer types and age categories (table 2).

However, there are differences between EHR systems used; MicroHis has the highest proportion of matching cases (64.5%, 534 out of 828) and the lowest proportion of potentially false-positive cases (35.5% 294 of 828). Promedico has the lowest proportion of matching cases (44.7%, 925 out of 2068) and the highest proportion of potentially false-positive cases (55.3%, 1143 of 2068).

**Figure 1:**
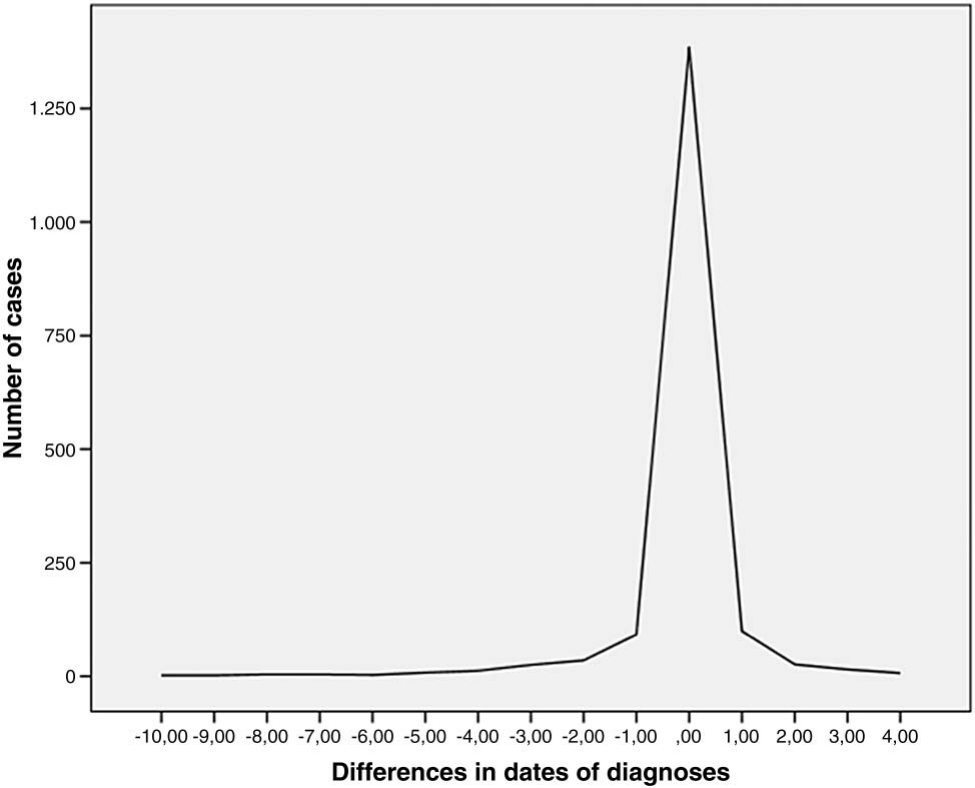
Deviation in the year of registered cancer diagnosis in the electronic health record from reference standard (Netherlands Cancer Registry).

The year of diagnosis in the EHR is registered in accordance with the NCR for 80.6% (1386 out of 1720) of cancer cases. For 75.5% (252 of 334) of cases with a different year of diagnosis, the deviation from the NCR incidence year is <2 years (figure 1).

Manual analysis of the full EHR text in a random sample of 120 unregistered NCR cases (potentially false-negative) shows that, even though these cases were not coded with an episode or journal consultation, for 29% (n=35) information about the cancer diagnosis is present in the EHR plain text, indicating the GPs’ awareness of the diagnosis. For another 23% (n=27), the cancer diagnosis is also mentioned, available in plain text, but the coding is based on cancer-related symptoms and not on the final cancer diagnosis (eg, breast cancer coded as ‘lump in the breast’ X19 and not recoded after confirmation of the diagnosis). In 17% (n=20) of cases, the GP registered a coded cancer diagnosis but added the date of registry (later than 2011 so not included in primary analysis) instead of the date of diagnosis. For 10 cases (8%), cancer was not found in our initial search (step 1 methods) but appears to be registered correctly when extracting the full EHR. This means that in 77% (29+23+17+8) of the cancer diagnoses qualified as potentially false negative in the EHR registries, the cancer diagnosis is actually known to the GP and can be recognised in the EHR with adequate text-finding strategies.

For another 8% (n=10), no information could be traced indicating the presence of cancer in the full-text EHR. For 2% (n=2), another valid explanation for the missing coded cancer diagnosis is present in the EHR text: one patient moved and unlisted with his GP and for another patient the diagnosis was made after death and the GP did not add this diagnosis to the EHR. For 13% (n=16), no written journal EHR data linked to the patient could be retrieved in the JGPN database.

Analysis of the full EHR text of another 120 randomly selected potentially incorrectly assigned cancer diagnoses shows that, for 18% of these seemingly false-positive diagnoses, clear and reliable indications of the presence of cancer in the EHR are found, while no diagnosis is present in the NCR. For 49% (n=59) of false-positive diagnoses, no reason can be traced in the EHR text (table 4).

**Table 4:** Causes of false-negative and false-positive registration of cancer diagnoses in the electronic health records

Number	Per cent	Comments
*False-positive diagnoses*		
**59**	**49**	**No explanation**
59	49	No logical reason can be traced in the full EHR text about the registration of a cancer code with this patient
**15**	**13**	**Coding error by GP**
15	13	Coding error by GP (eg, ‘R84’ lung cancer when lung cancer is suspected by the GP)
**46**	**38**	**Diagnosis correct in EHR**
16	13	Year of diagnosis in the EHR is 2011, leaving a small chance that the histological confirmation of the diagnosis (NCR) was performed (and registered) in 2012, while the clinical diagnosis was made and registered in the EHR in 2011
11	9	Year of diagnosis >10 years before 2007; cancer not available in the NCR
8	7	Cancer registered twice at GP
7	6	Patient has moved or diagnosis was made abroad, thus not in the NCR
4	3	No tissue biopsy was performed in agreement with patient and/or family, thus not in the NCR
**120**	**100**	
*False-negative diagnoses*		
**92**	**77**	**Information about cancer is available**
35	29	Information about cancer is available in plain text in the EHR but cancer is not coded
27	23	GP assigned a wrong code/did not update existing code after diagnosis (eg, ‘X19’ lump in breast instead of ‘X76’ breast cancer)
20	17	Cancer is coded in the EHR but GP assigned a year of diagnosis >2011
10	8	Coded cancer found in full EHR but patient was not in the initial search due to time lapse between the initial search and linkage (>1 year)
**16**	**13**	***EHR record cannot be retrieved***
16	13	Patient EHR cannot be retrieved (probably linkage error)
**10**	**8**	***No explanation***
10	8	No EHR text or codes about any cancer
**2**	**2**	***Various***
2	2	Remaining causes: patient has moved, diagnosis after death
**120**	**100**	

EHR, electronic health record; GPs, general practitioners; NCR, Netherlands Cancer Registry.

Reviewing these numbers, 90% of NCR-confirmed cancer cases can be recognised and found in primary care EHR systems, counting for two-thirds of the EHR-coded cancer cases. An additional 10% of coded cancer cases in primary care EHR systems should be considered correct, but stay unvalidated since the diagnosis did not reach the NCR for various reasons. Approximately 20% of cancer cases found in EHR systems should be considered to be wrongly coded, false-positive cases.

## Discussion

### Principal findings

Extracting coded cancer diagnoses from a primary care EHR (JGPN) and linking these to the NCR demonstrate matching diagnoses in over 60% of cases. Almost 40% of cancer cases registered in the NCR are missing in the EHR (table 2). However, for at least 77% of these false-negative coded diagnoses, uncoded information indicating the GPs’ knowledge of cancer can be found in the EHR (table 4). Overall, GPs seem to know the great majority of their patients with cancer, since 90% of the NCR-validated cancers are also described in EHR systems.

Almost half of the coded cancer diagnoses in the EHR seem to be false positive (table 2), of which only a minority can be explained by wrongly used diagnostic coding such as coding symptoms as actual cancer.

There are differences up to 20% in proportions of correct (matching), missing (false-negative) and wrong (false-positive) cancer diagnoses between EHR systems but not between age categories or cancer types. The year of diagnosis in the EHR is confirmed in over 80% of matching cases. For incorrectly registered diagnosis-years, 76% deviate no more than 2 years from the NCR.

### Strengths and weaknesses

To the best of our knowledge, this is the first study to use record linkage to assess quality of cancer registry in routine primary care data, combined with a search for actual causes of inadequate registry and resulting opportunities for improvement. The major strengths of this study are the size of the cohort, the extensive EHR data and the availability of a reliable reference standard (NCR). Furthermore, the JGPN database comprises unmanipulated EHR data as available from routine care, hence not improved or enriched in any way such as in the UK Clinical Practice Research Datalink, formerly known as General Practice Research Database.^23^

Our study has some restrictions. Since our study was performed in the Netherlands, the results are indicative for settings similar to ours, which means countries with the GP in a gatekeeper role, which have adapted to the use of EHRs in primary care resulting in relatively ‘mature’ EHR systems. We were able to analyse only a sample of false-positive and false-negative cases. In our study, a number of missing (false-negative) cases (21% (13+8) of our sample of 120) could not be traced in the JGPN, and no explanation regarding wrongfully coded cancer diagnoses could be found in 49% of false-positive cases.

Since no unique identifiers could be used for linkage, we used the commonly used alternative method of probabilistic linkage. Consequently, discrepancies in databases could in part be a consequence of linkage errors, which could have biased our results in either direction. The primary problem that may occur is the rare occurrence of matching two different patients with identical characteristics. This would result in the false assumption that a cancer diagnosis registered in the NCR is ‘missing’ in the matched patient in the EHR. This is expected to occur in <1% of cases. Another linkage error which may occur is ‘no match’ for a patient who is registered in both databases, but not by the same characteristics used for linkage. Since we used date of birth, gender, zip code, last name, initials and first name, these characteristics are unlikely to be registered differently in the registries. Only in case of typing errors, moving out of the zip code area or changing last names within the time frame between dates on which the data were extracted from the different databases, or in case of not registering such a previous change in one of these databases, such linkage error will occur. We estimate the chance of such an occurrence to be below 1%.

To calculate the concordance in year of diagnosis, we used the calendar year in which the diagnosis was registered. Consequently, in the EHR and the NCR, a diagnosis registered in January of a calendar year, for example, 2011, and a diagnosis registered in December of the same year are considered to be, for this example, ‘registered in 2011’. This means that a difference in registration of ‘1 year’ could be either 2 days (registered in JGPN on 31 December 2010 and in NCR on 1 January 2011) or nearly 2 years (registered in JGPN on 1 January 2010 and in NCR on 31 December 2011). Since we used this rough measure, we only showed the absolute numbers and refrained from providing statistical testing for concordance.

### Comparison with existing literature

Two Dutch^24^ ^25^ studies and one Swiss^26^ study investigating the coding of diagnoses in primary care EHRs concluded that the quality of coding in general was fairly good but varied widely between general practices. None of these studies used record linkage, and the largest study (1.1 million patients)^24^ assessed only the presence of ‘meaningful’ ICPC diagnostic codes in the EHR. In another Dutch study,^13^ the diagnosis inflammatory arthritis in Dutch EHRs could be validated in 71–78% of 219 patients by comparison with correspondence from a medical specialist.

In a UK Clinical Practice Research Datalink (CPRD) database study,^14^ a high (93%) positive predictive value of Read Codes for congenital cardiac malformations registered between 1996 and 2010 was found. However, 31% of cases had a different event date, including 10% that differed no more than 30 days. These results cannot readily be generalised because practices contributing data to the CPRD are accepted only when they meet standards of data completeness.^27^ Even though the five studies described so far assessed quality of non-cancer disease registry, they all present outcomes that are in line with our findings.

There are several studies that assess the quality of disease registry in primary care for cancer. Boggon *et al*^15^ reported a concordance level of 83.3% between CPRD records from a Diabetes cohort and the UK National Cancer Data Repository. This is higher than the proportion of matching cases (concordance level) at first sight of 60.6% that we found, but might be comparable to the 80% we found when using additional search techniques. Also the proportion of false positives (17%, 967 of 5.797) and false negatives (6%, 341 of 5.676) are much lower than the proportions we found, but again, only high-quality data are imported in the CPRD. Boggon *et al* also found relevant differences between cancer types and age categories (less concordance with increasing age), which we did not.

Pascoe *et al*^10^ recruited five GP centres for a retrospective analysis of EHR records on registration of cancer diagnoses compared to a regional cancer registry in the UK. One in five (20%) of all primary care patients with cancer was not identified when a search for all patients with cancer was conducted using codes for malignancy. Also 20% of patient records with a code for malignancy that was confirmed in the cancer registry lacked the necessary documentation to verify cancer documented in the EHR. Overall, codes for cancer in these EHRs had a poor level of completeness (29.4%) and correctness (65.6%), if compared to the UK Cancer Registry as the reference standard.

The California Kaiser Permanent study^28^ aimed to assess variability in date of prostate cancer diagnosis between 2000 and 2010 by comparing Cancer Registry, pathology reports and EHR data. Variability in date of diagnosis was found: from 9.6 years earlier to 10 years later, but the vast majority of deviations was small. These results are comparable with the results in our study, although our deviations ranged from −10 to+4 years. A recent study by Kearney *et al*,^29^ validating the completeness and accuracy of the Northern Ireland Cancer Registry (NICR), found a high level of completeness (99.9%) within the NICR compared to the GP registries. The authors suggest that these excellent results could be induced by the introduction of the National Health Service (NHS) unique identifier in 2008, which enables matching and data enrichment, but also by financially rewarding GPs who maintain a high-quality up-to-date record of patients with chronic diseases, including cancer.^29^ ^30^

### Meaning and implications for research and practice

Do GPs know their patients with cancer? Yes, our data show they do know the vast majority. Does this mean reusers of data can retrieve all these patients using coded diagnoses? No, because the proportion of wrong and missing diagnoses is too high. If reusers have access to full-text, they would be able to identify up to 90% of cases reliably using labour-intensive manual exploration or text-mining techniques of EHRs. Also, 20% of cases identified will have to be excluded after reassessment. For some purposes this will be acceptable, for other purposes it will not be.

Although we have shown that GPs seem to know their own patients with cancer, locums and doctors working at out-of-hours clinics do rely heavily on EHR data, including coded diagnoses. Missing (false-negative) and wrong (false-positive) cancer diagnoses on this list could have adverse effects on clinical practice, including medical decisions made elsewhere. Also, patients could perceive errors in diagnosis lists as unprofessional and unreliable. From a research perspective, erroneously including patients without cancer (false positives) and missing real cancer cases (false negatives) may introduce bias. If text-mining techniques are used, these results improve substantially, as was shown in this study as well as in others.^31^ However, the possibility of residual confounding cannot be completely excluded.

A number of causes for suboptimal registry have been demonstrated in our study that might be used as a starting point for improving data quality at the source, hence at data entry. Improvements could be made (1) through education for practising and future GPs, by improving usability of (2) EHR systems and (3) coding systems.

First of all, GPs' awareness and coding skills could possibly be improved through education in order to decrease coding errors and errors in the registered year of diagnosis. Although we have not found any studies proving education can actually improve data quality, we do know that financial incentives as well as feedback using data quality reports do improve data quality.^30^ ^32^ ^33^ This shows that improving registration quality is feasible and can be learnt. Furthermore, GPs could evaluate and update working processes at the GP practice to integrate diagnosis registry after a letter from a hospital or diagnostic laboratory is received.

Second, EHR systems could be improved by facilitating user-friendly and accurately coded diagnosis registry. Some systems are subject to less false-positive diagnoses and a higher number of accurate cancer diagnoses. Since we do not expect these differences to be caused by confounding resulting from different types of GPs choosing certain types of EHRs, this implies that differences in system design lead to varying data quality.

Adding options which are now missing, for example, to directly register date of diagnosis, suspected, recurring and metastasising disease, treatment, increased markers (eg, for Prostate Specific Antigen or PSA) and a positive family history could also improve quality.

Third, improvements could also be realised by adding codes to the coding system (eg, for recurring and metastasising disease), by providing adequate synonyms to improve findability of codes and by adding relevant crosslinks to facilitate data sharing between sources.

Another strategy, which might improve clinical practice, was used in our study. Linkage has its benefits, since^23^ multiple data sources are often complementary and taken together have added value, as was also demonstrated in our study. For current GP practice, the effort that has to be put into requesting and performing the actual linkage process (including patient informed consent) is not worth the benefit. However, if structural linkage of EHRs to accurate medical data sources (such as the NCR) should become available, this information could be used to proactively alert and inform the GP to check and adjust recordings in routine care data.

For research purposes and quality assessment, we feel that validation of disease cases and/or improvement of EHR data is necessary before working with the data, particularly if only coded data are used. Research using EHR data provides access to a very rich data source, but its interpretation and use should only be performed in cooperation with experienced clinicians who can judge the meaning of the information in its context. Linkage could be one of the tools to decrease the number of false-negative coded cancer diagnoses. For a lot of diseases, however, no reference standard is available. Linkage to hospital records could improve data also by decreasing false-negative cases, but the quality of these data is also likely to be suboptimal. Studying the full EHR, which is time-consuming, might also be supported by automatised text-mining techniques which will help in identifying false-positive records. If in the future these techniques can be made more advanced and more reliable, these might replace the need for manual searching.

### Unanswered questions and future research

Future research should, besides correctness and completeness, evaluate other dimensions of data quality; concordance, plausibility and currency.^9^ Also, quality of other key data items besides the diagnosis should be studied, for instance risk factors, treatment and allergies. In cooperation with the NCR, further exploration of the cases where the EHR seems to provide reliable indications of the presence of cancer, whereas there is no record in the NCR, is needed in the near future.

Evaluating the user interface of the various EHR systems and determining how these explain the differences in data quality in this study would be a worthwhile exercise.

Furthermore, in this study we investigated a serious and relatively common disease; investigating the quality of registry for more rare or less serious diseases may provide different results, which would be of additional value.

Last but not least, the design, implementation and evaluation of actual interventions in the GP practice to improve disease registry would provide a necessary next step in improving EHR data quality. Improving text-mining software and strategies could be part of this.

## Concluding remarks

Yes, GPs do know the vast majority of their patients with cancer. However, reusers of coded Electronic Healthcare data should be aware that they are at risk of missing 40% of cancer cases and that almost half of the cancer cases found could be wrongfully registered. Analysing the full-text EHRs improves these numbers: only 10% of cases will be missed and 20% of cases found will be wrong. Particularly in non-clinical circumstances like research, when high accuracy is needed, primary care EHR data should only be reused with care.
